# Knowledge and Awareness of Sickle Cell Disease Among Premarital Screening Participants in Makkah, Saudi Arabia: A Cross-Sectional Study

**DOI:** 10.7759/cureus.106766

**Published:** 2026-04-10

**Authors:** Ibrahim Alharbi, Badr S Alsaeedi, Khalid Alsaedi, Feras Alwadani, Yasir Almohammadi, Abdulaziz Alshinqiti, Suliman Aloyoni

**Affiliations:** 1 Department of Pediatrics, Faculty of Medicine, Umm Al-Qura University, Makkah, SAU; 2 Department of Medicine and Surgery, Faculty of Medicine, Umm Al-Qura University, Makkah, SAU

**Keywords:** awareness, cross-sectional study, knowledge, premarital screening, sickle cell disease

## Abstract

Introduction

Sickle cell disease (SCD) is one of the most common hereditary blood diseases in Saudi Arabia. A premarital screening program is obligatory to be done before couples get married in Saudi Arabia. Our aim in this study is to assess the level of knowledge and awareness of SCD among individuals performing the premarital screening program in Makkah city.

Methods

This cross-sectional study was conducted among 390 adults in Makkah city, Saudi Arabia, who were planning to get married, from August to December 2025. The electronic questionnaire collected sociodemographic data, as well as knowledge and awareness regarding the premarital screening program for SCD.

Results

A total of 390 participants participated, with the majority aged 18-29 years (51.0%), male (66.2%), single (75.1%), urban inhabitants (95.1%), and bachelor's degree holders (59.2%). Even while 69.7% of respondents had heard of SCD, only 23.6% of them showed a strong understanding, and 76.4% had poor overall knowledge. Although 62.3% acknowledged blood testing as a diagnostic tool and 57.2% recognized SCD as hereditary, there were significant gaps in knowledge regarding inheritance risk, complications (63.1% insufficient knowledge), and crisis management (61.3% unaware). The majority of individuals thought premarital screening lowers the probability of the disease (68.2%) and were aware that it included SCD testing (68.7%). If both couples were carriers, the majority (86.4%) were against marriage. Better knowledge was substantially correlated with higher education (p=0.025), marital status (p=0.025), female gender (p=0.013), and healthcare practitioners as an information source (p=0.001).

Conclusion

Despite positive attitudes toward premarital screening in Makkah city, participants showed poor knowledge of SCD, especially regarding inheritance, complications, and management. Awareness was higher among women, married individuals, the highly educated, and those informed by healthcare providers. Standardized counseling and targeted education are essential to improve understanding of genetic risks.

## Introduction

Sickle cell disease (SCD) is one of the most common hereditary blood diseases in Saudi Arabia. The disease is considered a multisystem disease; it is an autosomal recessive disorder of the blood hemoglobin, affecting the amino acid sequence and leading to the substitution of glutamic acid by valine. The glutamic acid is usually located in the sixth position of the beta chain of the hemoglobin [1]. SCD can present in a variety of symptoms ranging in severity. It is mainly caused by the abnormal sickling of the blood, which leads to microcirculation complications such as vaso-occlusive crisis, chronic hemolytic anemia, and infection due to auto-splenectomy, among other serious complications [2]. Hemoglobinopathies such as “thalassemia” and “SCD” are globally prevalent diseases, which account for about 5% of the world population, and are very common in countries in sub-Saharan Africa, India, Saudi Arabia, and Mediterranean countries [3].

The premarital screening program is defined as a set of tests conducted for individuals planning to get married. These tests include genetic screenings (e.g., for SCD and thalassemia) and infectious disease screenings (e.g., for hepatitis B/C and HIV/AIDS) [4]. In 2022, a cross-sectional study was done to measure the level of public knowledge, awareness, and attitude regarding premarital screening for SCD and thalassemia. A total of 374 people in Makkah city answered a survey, which showed that 99.2% support the screening program. Around 51.1% of the answers showed good knowledge, and the results also showed that awareness was very high in married people, university-educated individuals, and older age groups [5]. A cross-sectional study was done in 2023 on the general population of Saudi Arabia regarding knowledge and attitudes toward SCD and premarital screening. Seven hundred and eighty-nine people answered the questionnaire, and only 10% had good knowledge of SCD. On the other hand, 74.9% had moderate knowledge and 15.1% had poor knowledge. Furthermore, 34.7% had a positive attitude regarding the premarital screening program and SCD, while 64.9% had a neutral attitude [6]. In the previous year (2024), a cross-sectional study was done in the Al-Baha region. A survey was conducted with 478 participants regarding public awareness and practices of premarital screening for SCD. The study revealed that only 50% of married or engaged people had taken the premarital screening test. A total of 66.2% reported consanguinity as a known risk factor for SCD. Awareness of SCD and the premarital screening program was low, with only 59.8% of participants having heard of SCD and 63% aware of the screening program [7]. Despite several studies conducted in Saudi Arabia, these studies have not addressed this issue specifically in Makkah city. Additionally, they have not concentrated on a particular target group, like individuals who will take part in the premarital screening program. Because of this, we are conducting this study to address the lack of current data on knowledge and awareness of this issue among the Makkah population. Therefore, our aim in this study is to assess the level of knowledge and awareness of SCD among individuals attending the premarital screening program in Makkah city. Moreover, the study aims to identify sociodemographic and informational factors associated with knowledge levels in this population.

## Materials and methods

Study design

For this study, a cross-sectional design was used, as it is a descriptive epidemiological study aimed at assessing knowledge and awareness of premarital screening for sickle cell anemia among adults about to get married in Makkah city, Saudi Arabia.

Study setting

The study included the adult population of the city of Makkah, Saudi Arabia, located in the western region of the Kingdom of Saudi Arabia, specifically in the Hejaz region, within the Tihama Plain near the Red Sea coast. The target population for this study was individuals aged 18 or older of both genders who were about to get married and were selected from the city of Makkah using a convenience sampling method. The study was conducted over five months, from August to December 2025.

Study population

The number of adults aged 18 or older residing in Makkah city has exceeded two million, according to the General Authority for Statistics [8], and the study will target 390 participants who are about to get married.

Eligibility criteria

Inclusion Criteria

Males and females aged 18 years, who were about to get married, who could communicate in Arabic or English, and who were willing to participate in the study were included.

Exclusion Criteria

Participants who were not able to provide informed consent or complete the study questionnaire, or who did not reside in Makkah city, were excluded.

Sample size and sampling technique

The target population for this survey included all adults aged 18 or older who were about to get married in the city of Makkah and were selected by the city’s government using convenience sampling. The sample size for this study was determined using the statistical formula: $n = \frac{Z^2 \times p \times (1 - p)}{e^2}$.

The formula parameters included n: sample size, z: standard normal distribution (1.96 at a 95% confidence level), p: anticipated population proportion, q: 1-p, and e: margin of error. The anticipated population proportion (p) of the sample was estimated at 50% because this is the safest option, as the required sample size is largest when p = 50%. It was assumed that half of the population had relatively low knowledge and awareness of premarital screening for sickle cell anemia; therefore, p = 0.5. With a 95% confidence level and a 5% margin of error, the sample size for this study required 390 adult participants who were about to get married. Participants were invited to participate in the study by following the survey link. Copies of the survey were distributed through links and QR codes placed in premarital screening centres and via official marriage registrars.

Data collection tools

A structured, self-administered online questionnaire measuring knowledge and awareness about SCD and premarital screening was used to gather data. The questionnaire was constructed based on a review of previously published studies. To guarantee content validity, the questionnaire was examined by one faculty member with backgrounds in hematology and public health. Twenty individuals participated in a pilot study to evaluate readability, clarity, and completion time; small adjustments were made in response.

The questionnaire consisted of 20 closed-ended questions divided into four sections: informed consent, socio-demographic characteristics, knowledge and awareness of SCD, and knowledge of premarital screening (Appendices).

Single-answer questions were scored 1 for a correct answer, 0 for an incorrect answer, or 0 for “I don’t know.” For multiple-answer questions, each correct answer was scored, and incorrect answers were not counted. A total knowledge score was calculated, with higher scores indicating greater awareness.

Bias

There are several potential causes of bias in this cross-sectional research. These include the possibility of selection bias brought on by the use of voluntary participation and convenience sampling, which might reduce the research population's representativeness. The fact that some people volunteered because they were interested in this topic raises the possibility of non-response bias. The use of self-reported data, which might be impacted by incorrect answers or misinterpretations of the questions, may have resulted in information bias. Participants may have given responses deemed socially acceptable due to social desirability bias, especially for issues pertaining to attitude and practice. Recall bias might possibly have influenced replies to earlier diagnoses or personal medical history.

Data management (collection process)

After obtaining ethical approval to conduct the study among adults about to get married in Makkah city, the online questionnaire was shared to collect data.

Identification

Adults who were about to get married in the city of Makkah were identified, excluding those mentioned in the exclusion criteria.

Approach

After identifying study participants and assessing inclusion criteria, participants who met the criteria were approached and informed of the study’s principles. Those interested in participating were asked to provide their consent by selecting “agree” or “disagree” in the checkbox.

Recruitment

Interested participants were recruited. Recruitment included completing an online survey via the survey link.

Handling Missing Data

Four hundred and twenty questionnaires were distributed, and 30 responses were excluded (about 7%) because incomplete or missing information was unsuitable for inclusion. That left 390 completed answers for the analysis. Finally, only completed questionnaires were used to maintain the quality and accuracy of the results.

Data pilot

A pilot study was conducted with 20 participants to test the questionnaire’s validity, question clarity, and completion time. This number was not added to the study cases.

Ethical considerations

Ethical approval was obtained from the Research Ethics Committee at Umm Al-Qura University. All necessary official permits were obtained before any data collection. Informed consent was obtained from all participants in this study. Data confidentiality was ensured. Data collected from adults were used solely for scientific purposes.

Funding

The researchers bore all costs of this study.

Data analysis

Data were entered, coded, and analyzed using the Statistical Package for the IBM SPSS Statistics for Windows, Version 28 (Released 2021; IBM Corp., Armonk, New York, United States). Descriptive statistics were used to record the participants' socio-demographic traits, information sources, knowledge items, and attitudes towards SCD and premarital screening. Categorical variables were presented using frequencies and proportions. The overall knowledge and awareness of SCD were evaluated using a structured scoring system. Correct answers to knowledge-related questions earned one point, while incorrect or "I don't know" responses were given zero points. A percentage was determined by calculating and converting an individual's total knowledge score. Those who scored below 60% were considered to have poor knowledge and awareness. In contrast, those with a score of 60% or higher were considered to have good knowledge and awareness.

Inferential statistical analysis was conducted to examine associations between participants’ knowledge and awareness levels and selected socio-demographic characteristics, sources of information, and attitude variables. The Pearson chi-square test was used to assess associations between categorical variables, and the exact probability test was used when the chi-square test assumptions were not met. All statistical tests were two-tailed, and a p-value of less than 0.05 was considered statistically significant. No sensitivity analysis was conducted, as the analysis was based on complete cases and predefined scoring criteria.

## Results

Table 1 summarizes the demographics of the 390 participants who were enrolled in the premarital screening program in Makkah city. Participants were more likely to be 18-29 years (199; 51.0%) than those aged 30-44 years (140; 35.9%), and the proportions were observed in older age groups (45-59 years: 10.5%; ≥60 years: 2.6%). Nearly two-thirds of the sample (258; 66.2%) were males. Singles made up the majority of participants (293; 75.1%), with married individuals making up 24.9% (97). Urban areas (371; 95.1%) were the most common place for respondents to reside, with only a small proportion living in rural areas (19; 4.9%). The number of participants with a bachelor's degree was over half (231; 59.2%), while 111 (28.5%) had secondary education or lower, 15 (3.8%) had a diploma, and 33 (8.5%) had postgraduate qualifications.

**Table 1:** Sociodemographic Characteristics of Study Participants in the Premarital Screening Program in Makkah City (N = 390)

Sociodemographic data	N	%
Age in years		
18-29	199	51.0
30-44	140	35.9
45-59	41	10.5
60	10	2.6
Gender		
Male	258	66.2
Female	132	33.8
Marital status		
Single	293	75.1
Married	97	24.9
Residence area		
Urban	371	95.1
Rural	19	4.9
Educational level		
Secondary/below	111	28.5
Diploma	15	3.8
Bachelor degree	231	59.2
Postgraduate	33	8.5

Table 2 shows the distribution of participants' knowledge and awareness of SCD among those attending the premarital screening program in Makkah city. Overall, SCD was reported to have been previously heard about by 272 participants (69.7%). Regarding the causes of SCD, more than half of the respondents correctly identified heredity as the main cause (223; 57.2%), while smaller proportions attributed the disease to immune disorders (27; 6.9%), poor lifestyle (8; 2.1%), poor nutrition (12; 3.1%), infection (4; 1.0%), or environmental pollution (3; 0.8%). Concerning the epidemiology of SCD, half of the participants (196; 50.3%) recognized that the disease is more common in communities with a high rate of consanguineous marriage, whereas 48 (12.3%) believed there was no relationship, and 11 (2.8%) linked it to low consanguinity. Knowledge of SCD symptoms varied, with fatigue and exhaustion (148; 37.9%) and skin pallor (128; 32.8%) being the most frequently identified symptoms. Other reported symptoms included headache (67; 17.2%), recurrent infection (59; 15.1%), visual problems (54; 13.8%), vomiting (32; 8.2%), and diarrhea (19; 4.9%). Knowledge of SCD complications was limited. Occlusion of blood vessels was identified by 63 participants (16.2%), followed by chronic infection and hypertension (62; 15.9%), stroke (45; 11.5%), reduced blood sugar (37; 9.5%), leg ulcers (30; 7.7%), and increased thyroid activity (24; 6.2%). There was a significant number of respondents (246; 63.1%) who reported not being aware of any complications. A total of 243 participants (62.3%) correctly identified blood tests as diagnostic, with fewer participants choosing X-rays (11; 2.8%), electrocardiography (4; 1.0%), or intestinal endoscopy (2; 0.5%). The lack of knowledge about genetic transmission was significant, as only 94 participants (24.1%) correctly identified the 100% risk of having an affected child when both parents are affected. Other individuals chose probabilities that were not accurate, such as 75% (52; 13.3%), 50% (51; 13.1%), 25% (20; 5.1%), or 0% (3; 0.8%). The ability to manage crises was limited, with only 36 participants (9.2%) accurately identifying the need for sufficient fluids, painkillers, and oxygen therapy. In total, 115 participants selected other options, whereas the majority (239; 61.3%) reported not knowing the appropriate management. Regarding cureability, there was uncertainty: 142 participants (36.4%) believed there might be a permanent cure, 74 participants (19.0%) reported that there is no permanent cure, and 57 participants (14.6%) believed there is no cure.

**Table 2:** Participants’ Knowledge and Awareness of Sickle Cell Disease in Makkah (N = 390)

SCD Knowledge		N	%
Have you ever heard about sickle cell disease?	Yes	272	69.7
	No	118	30.3
The causes of sickle cell disease	Hereditary	223	57.2
	Immune disease	27	6.9
	Poor lifestyle	8	2.1
	Poor nutrition	12	3.1
	Infection	4	1.0
	Environmental pollution	3	0.8
	I don't know	113	29.0
Sickle cell disease is more common in	A community with a high rate of consanguineous marriage	196	50.3
	A community with a low rate of consanguineous marriage	11	2.8
	No relation between sickle cell disease and consanguineous marriage	48	12.3
	I don't know	135	34.6
Symptoms of sickle cell disease	Skin pallor	128	32.8
	Fatigue and exhaustion	148	37.9
	Diarrhea	19	4.9
	Visual problem	54	13.8
	Vomiting	32	8.2
	Recurrent infection	59	15.1
	Headache	67	17.2
	I don't know	196	50.3
Complication of sickle cell disease	Chronic infection	62	15.9
	Stroke	45	11.5
	Elevation of blood pressure	62	15.9
	Ulcers of the legs	30	7.7
	Reducing blood sugar	37	9.5
	Occlusion of blood vessels	63	16.2
	Increase thyroid activity	24	6.2
	I don't know	246	63.1
How is sickle cell disease diagnosed?	Blood test	243	62.3
	X-ray	11	2.8
	Electrocardiography	4	1.0
	Intestinal scope	2	0.5
	I don't know	130	33.3
What is the percentage of having a child with sickle cell if both parents are diseased?	100%	94	24.1
	75%	52	13.3
	50%	51	13.1
	25%	20	5.1
	0%	3	0.8
	I don't know	170	43.6
The management of sickle cell crisis	Giving the patient a large amount of fluids, painkillers, and oxygen therapy	36	9.2
	Fever-reducing medicine, along with giving the patient a large amount of fluids	31	7.9
	Giving the patient a large amount of fluids and painkillers	67	17.2
	Fever-reducing medicine and painkillers	17	4.4
	I don't know	239	61.3
Do you think there is a permanent cure for sickle cell disease?	Yes	57	14.6
	No	74	19.0
	Maybe	142	36.4
	I don't know	117	30.0

Table 3 presents participants' awareness and attitudes towards premarital screening and its ability to prevent SCD. More than two-thirds of respondents (268; 68.7%) were aware that SCD testing is part of premarital screening, with only a few (24; 6.2%) believing otherwise, and approximately three-quarters (98; 25.1%) were unsure. Regarding the preventive role of premarital screening, more than two-thirds of participants (266; 68.2%) agreed that it reduces the incidence of SCD, whereas 36 participants (9.2%) disagreed and 88 (22.6%) were not sure. When asked about their attitude toward marriage if both partners are carriers of SCD, the vast majority of respondents (337; 86.4%) opposed proceeding with the marriage, whereas a small minority (53; 13.6%) supported it.

**Table 3:** Participants’ Awareness and Attitudes Toward Premarital Screening and Its Role in the Prevention of Sickle Cell Disease in Makkah City (N = 390)

Items	N	%
Do you think premarital screening includes sickle cell diseases?		
Yes	268	68.7
No	24	6.2
Maybe	98	25.1
Do you think premarital screening plays a role in reducing the incidence of sickle cell disease?		
Agree	266	68.2
Disagree	36	9.2
Not sure	88	22.6
If it is found that both parents are carriers of sickle cell disease, do you support the marriage?		
Agree	53	13.6
Disagree	337	86.4

**Figure 1:**
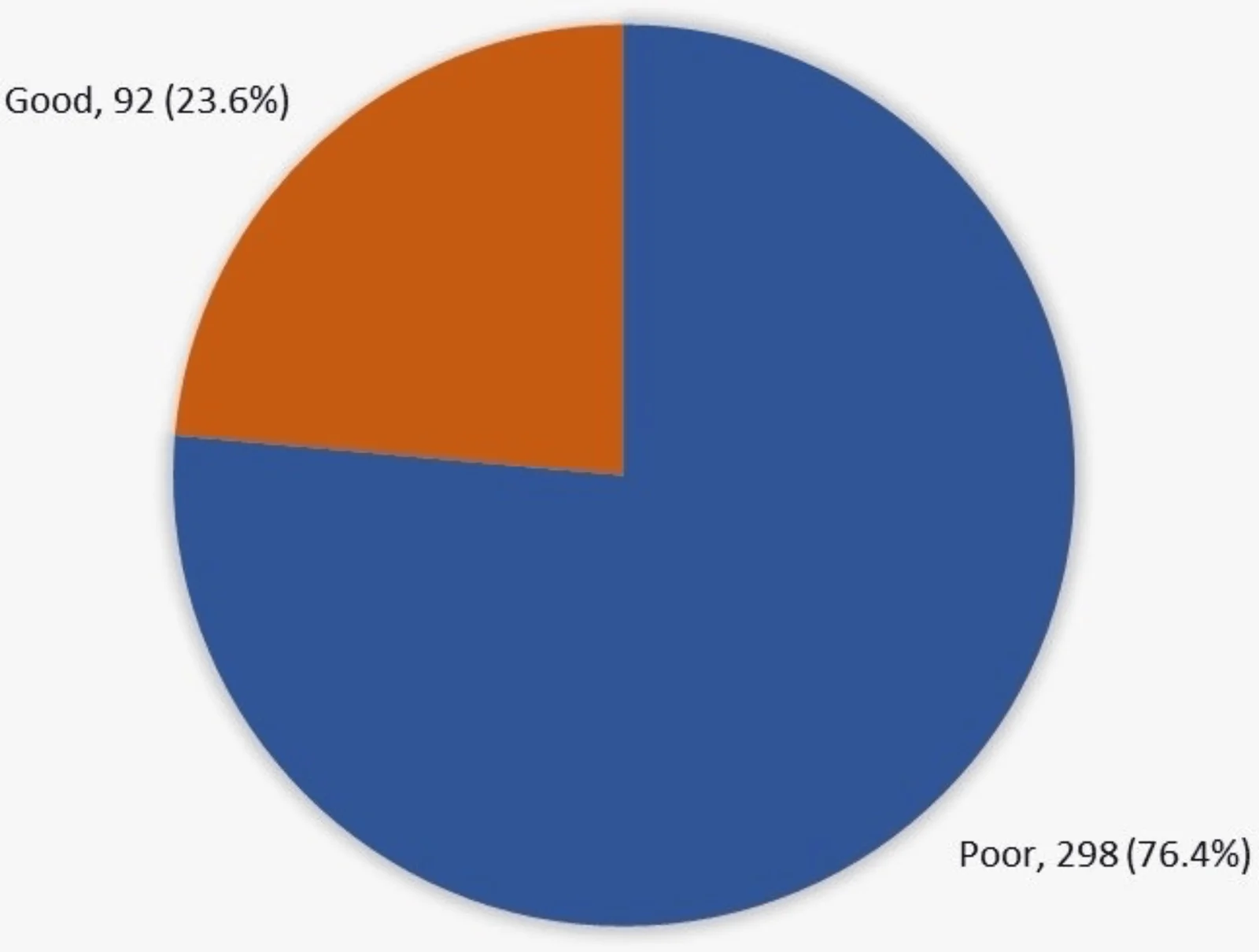
Overall Participants’ Knowledge and Awareness of Sickle Cell Disease in Makkah (N = 390)

**Figure 2:**
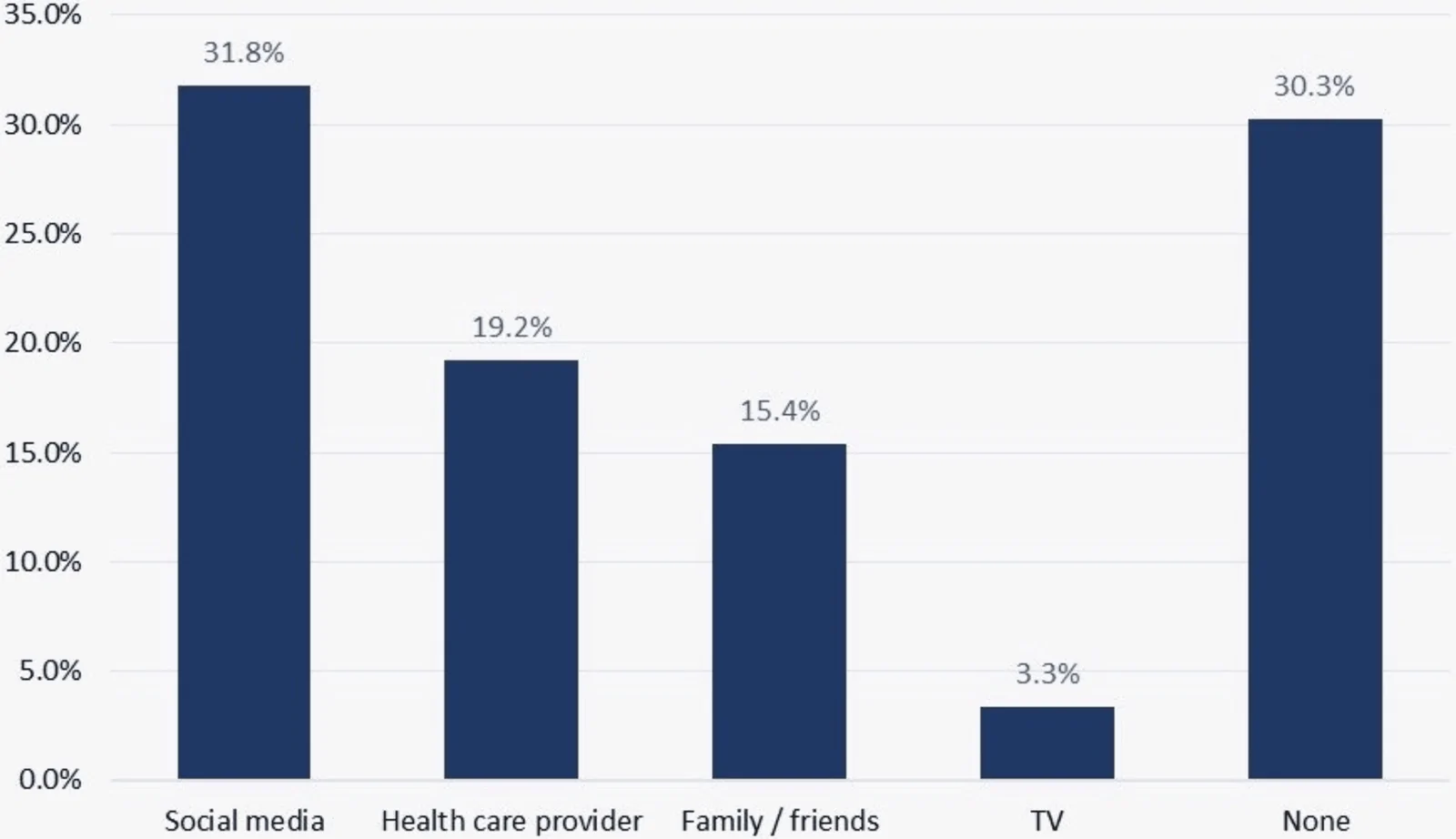
Sources of Participants’ Information About Sickle Cell Disease in Makkah (N = 390)

Figure 1 shows that the overall level of knowledge and awareness about SCD among participants was poor, as reported by 298 individuals (76.4%), while fewer than one quarter of the sample showed a good level of knowledge and awareness (92; 23.6%). Figure 2 shows that social media was the most common source of information (124; 31.8%), followed by healthcare providers (75; 19.2%) and family or friends (60; 15.4%). The percentage of participants who relied on television was lowest (13; 3.3%), but a significant segment of the sample reported having no sources of information (118; 30.3%).

Table 4 presents the factors associated with participants’ overall knowledge and awareness levels regarding SCD. Age was not significantly associated with knowledge level (p = 0.333), as poor knowledge predominated across all age groups, including all participants aged 60 years and above (10; 100.0%). Females had a higher proportion of good knowledge (41; 31.1%) than males (51; 19.8%), with a statistically significant association between gender (p = 0.013). Marital status was also significantly related to knowledge level (p = 0.025), as married participants showed better awareness (31; 32.0%) than single participants (61; 20.8%). Although urban residents had a higher proportion of good knowledge compared with rural residents (24.3% vs. 10.5%), the association between residence area and knowledge level did not reach statistical significance (p = 0.169). Educational level showed a significant positive association with knowledge and awareness (p = 0.025), with the proportion of good knowledge increasing progressively with higher educational attainment, reaching its highest level among participants with postgraduate education (13; 39.4%). The source of information appeared as a strong determinant of knowledge level (p = 0.001). Those who mentioned healthcare providers as their source of information had the highest percentage of good knowledge (36; 48%), while those who reported no source of information had overwhelmingly poor knowledge levels (115; 97.5%).

**Table 4:** Factors Associated With Participants’ Knowledge and Awareness of Sickle Cell Disease in Makkah (N = 390) χ^2^: Pearson chi-square test; ^: exact probability test (EPT); * P < 0.05 (significant)

Factors	Overall Knowledge and Awareness Level				χ^2^; p-value
	Poor		Good		
	N	%	N	%	
Age in years					
18-29	149	74.9	50	25.1	χ^2^ = 2.15; 0.333
30-44	108	77.1	32	22.9	
45-59	31	75.6	10	24.4	
60	10	100.0	0	0.0	
Gender					
Male	207	80.2	51	19.8	χ^2^ = 6.15; 0.013*
Female	91	68.9	41	31.1	
Marital status					
Single	232	79.2	61	20.8	χ^2^ = 5.02; 0.025*
Married	66	68.0	31	32.0	
Residence area					
Urban	281	75.7	90	24.3	EPT; 0.169^
Rural	17	89.5	2	10.5	
Educational level					
Secondary/below	94	84.7	17	15.3	χ^2^ = 8.20; 0.025*
Diploma	12	80.0	3	20.0	
Bachelor degree	172	74.5	59	25.5	
Postgraduate	20	60.6	13	39.4	
Source of information					
Healthcare provider	39	52.0	36	48.0	χ^2^ = 20.7; 0.001*
Social media	92	74.2	32	25.8	
Family/friends	42	70.0	18	30.0	
TV	10	76.9	3	23.1	
None	115	97.5	3	2.5	

Table 5 reveals the association between participants’ attitudes toward premarital screening and their overall knowledge and awareness level about SCD. A statistically significant association was observed between knowledge level and awareness that premarital screening includes SCD (p = 0.001). Among participants with good knowledge, the vast majority recognized that premarital screening includes SCD (87; 94.6%), compared with a lower proportion among those with poor knowledge (181; 60.7%), while uncertainty was markedly higher in the poor-knowledge group (95; 31.9% vs. 3; 3.3%). Similarly, a significant association was found between knowledge level and belief in the role of premarital screening in reducing the incidence of SCD (p = 0.001). Participants with good knowledge were more likely to agree with this preventive role (82; 89.1%) compared with those with poor knowledge (184; 61.7%), whereas indecision was more common among participants with poor knowledge (82; 27.5% vs. 6; 6.5%). In contrast, attitudes toward supporting marriage when both partners are carriers of SCD did not differ significantly by knowledge level (p = 0.601). Disagreement with proceeding with marriage was high in both groups, reported by 256 participants (85.9%) with poor knowledge and 81 participants (88.0%) with good knowledge.

**Table 5:** Association Between Participants’ Attitudes Toward Premarital Screening and Their Overall Knowledge and Awareness of Sickle Cell Disease in Makkah City (N = 390) χ^2^: Pearson chi-square test; ^: exact probability test (EPT); * P < 0.05 (significant)

Attitude	Overall Knowledge and Awareness Level				χ^2^; p-value
	Poor		Good		
	N	%	N	%	
Do you think premarital screening includes sickle cell diseases?					
Yes	181	60.7	87	94.6	EPT; 0.001*^
No	22	7.4	2	2.2	
Maybe	95	31.9	3	3.3	
Do you think premarital screening plays a role in reducing the incidence of sickle cell disease?					
Agree	184	61.7	82	89.1	χ^2^ = 31.4; 0.001*
Disagree	32	10.7	4	4.3	
Not sure	82	27.5	6	6.5	
If it is found both parents are carriers of sickle cell disease, do you support the marriage?					
Agree	42	14.1	11	12.0	χ^2^ = 1.03; 0.601
Disagree	256	85.9	81	88.0	

## Discussion

This study assessed the knowledge, awareness, and attitudes toward SCD among individuals attending the premarital screening program in Makkah city. Overall, the findings revealed that, despite relatively high educational levels and widespread exposure to premarital screening services, considerable gaps persist in essential knowledge about SCD causes, inheritance, symptoms, complications, and management.

In the current study, most participants were young adults, mostly male, single, urban residents, and university-educated. This demographic profile is comparable to that reported in other Saudi premarital screening studies, reflecting the age group most likely to undergo mandatory premarital testing in the Kingdom [9,10]. The relatively high educational level observed might be expected to translate into better health literacy; however, the findings indicate that general education alone does not ensure adequate disease-specific knowledge, particularly for genetic disorders such as SCD.

Although about two-thirds of participants reported having heard about SCD, detailed knowledge was limited. Similar findings have been reported in other regions of Saudi Arabia. For example, in the Eastern Province, where awareness of SCD inheritance is usually high, about two-thirds of adults lacked detailed knowledge beyond basics [11]. On the other hand, in Jeddah, only 51.4%, about half of the participants, demonstrated a good understanding of SCD and complications, showing moderate awareness with some knowledge gaps [12]. In Albaha, over two-thirds knew basic features, but many had misconceptions about triggers and inheritance patterns [13]. This unsatisfactory level revealed a missed opportunity within premarital screening programs to reinforce structured health education.

More than half of the participants correctly identified SCD as a hereditary disease, consistent with findings from studies conducted across many Saudi regions [9,14,15]. However, a considerable proportion attributed SCD to incorrect causes such as immune disorders, infection, or lifestyle factors, indicating misconceptions that have also been documented in studies from Nigeria in Africa and other Middle Eastern countries [16-18]. Due to misunderstandings, informed reproductive decision-making may be challenged, and clearer communication about genetic inheritance during counseling sessions is needed.

There is a lack of certainty about the connection between SCD and consanguinity. Only 50% of participants acknowledged that SCD is common in communities with high rates of consanguinity. The finding is concerning, given the well-established role of consanguineous marriages in increasing the prevalence of autosomal recessive disorders in Saudi Arabia [19,20]. Comparable studies have reported higher recognition of this association, revealing regional variability in the effectiveness of public health education [9,21].

Awareness of SCD symptoms was generally poor, with fatigue and pallor being the most frequently identified manifestations. Pain crises, which are the hallmark clinical feature of SCD, were poorly identified. Similar deficiencies in symptom recognition have been reported among premarital screening attendees and even among caregivers of patients with SCD [22,23]. Although almost all participants in a survey of young adults in Lagos, Nigeria, had heard of SCD, only slightly over half had a good overall understanding of the disease, indicating a lack of understanding beyond basic awareness [24]. Public support for preventive strategies and long-term disease management programs may be reduced due to these gaps.

Blood testing was correctly identified by most participants when it came to diagnosis, which shows that they are reasonably aware of the role of laboratory investigations. This finding is consistent with studies showing that diagnostic aspects of premarital screening are better understood than disease mechanisms or outcomes [9,10]. However, the selection of inappropriate diagnostic methods by some respondents indicates that there is still confusion that needs to be addressed with standardized counseling materials.

The understanding of genetic transmission was significantly limited. Only a minority correctly recognize that if both parents are affected by SCD, all offspring will inherit the disease. Similar misconceptions regarding inheritance probabilities have been widely reported in Saudi Arabia and internationally [15,25]. Inaccurate understanding of genetic risk can lead to inappropriate reassurance or undue anxiety, underscoring the importance of clear, simplified explanations during premarital counseling.

Most participants struggled to identify appropriate measures such as hydration, analgesia, and oxygen therapy due to their lack of awareness about acute crisis management. The finding is based on reports from community-based studies in Africa and the Middle East, where SCD management is often misunderstood outside of healthcare settings [17,26]. Improving public knowledge of crisis management may enhance timely care-seeking and community support for affected individuals.

Perceptions regarding curability revealed substantial uncertainty. Only a small portion of individuals correctly stated that there is no permanent cure, even though advances like hematopoietic stem cell transplantation (HSCT) are limited to selected cases. In health education initiatives, it is important to emphasize the distinction between supportive treatment, disease-modifying therapy, and curative options. For example, the current non-curable but effective in reducing complications for SCD treatment options are hydroxyurea, which induces fetal hemoglobin synthesis, thus decreasing vaso-occlusive events and improving quality of life. Chronic red blood cell transfusions are used in stroke prophylaxis and symptomatic relief, but have risks of alloimmunisation and iron overload.

Many of our participants are unaware of available curative treatment options for SCD, including HSCT, which will result in disease-free survival exceeding 90% if there is a human leukocyte antigen (HLA) matched donor [27]. Its success is dependent on donor availability. However, it has risks for graft versus host disease, toxicity, and its high financial cost, in addition to hospitalization and post-treatment care, making it hard to access for many patients. Recently emerging is the option of gene therapies that directly target the gene basis of SCD. Lentiviral vector-mediated gene addition introduces a functional β-globin gene into autologous hematopoietic stem cells, enabling production of anti-sickling hemoglobin variants. CRISPR-Cas9-based genome editing allows for the precise disruption of genetic elements that suppress HbF expression or the direct correction of the sickle mutation, resulting in the restoration of normal hemoglobin production. The promise of durable clinical benefits is offered with these novel approaches that circumvent immunologic complications inherent to allogeneic transplantation [27,28].

Fortunately, most participants were aware that premarital screening includes SCD testing and believed it helps reduce disease incidence. The positive attitude is in line with earlier studies in Saudi Arabia that show that premarital screening is widely accepted as a public health measure [9,14]. Moreover, strong opposition to marriage when both partners are carriers indicates a generally preventive attitude, although cultural, emotional, and religious factors may also influence such decisions beyond knowledge alone. For example, a cross-sectional study that included 386 Saudi couples who had undergone premarital screening in Jazan province found that some were genetically incompatible for SCD or β-thalassemia. Despite this, factors influencing their decision to proceed with marriage, despite awareness of the risk of having affected offspring, included the belief that God determines any illness, the relationship between the couple, knowing couples with inherited blood diseases who have healthy children, perceiving the chance of transmission as low, planning to undergo in vitro fertilization, lack of confidence in counseling, financial arrangements completed before screening, fear of inability to marry a healthy partner, family pressure, plans to avoid having children, and avoiding loneliness [29]. These were the reasons they proceeded with marriage despite knowing the associated risks.

The overall poor level of knowledge observed in this study is consistent with findings from multiple Saudi regions [9,15]. Social media was reported as the most common source of information, while healthcare providers were associated with significantly better knowledge levels. Female gender, marital status, higher educational level, and receiving information from healthcare providers were significantly associated with better knowledge. These associations have been repeatedly documented in the literature and likely reflect greater health-seeking behavior and exposure to counseling among these groups [20,25]. In contrast, age and residence were not significantly associated with knowledge level.

From the point of view of public health, our results emphasize the necessity of enhancing premarital screening programs by adding organized educational activities in addition to laboratory testing. In particular, when it comes to genetic inheritance, medical complications, and crisis management, healthcare practitioners should be at the forefront of providing clear and consistent guidance. Since social media was found to be a significant information source, focused digital health campaigns could be an effective tool for raising public awareness and correcting misconceptions. Additionally, including culturally sensitive counseling techniques in premarital counseling may improve comprehension and assist couples in making well-informed decisions. To increase the overall efficacy of SCD prevention programs, national initiatives should concentrate on standardizing counseling protocols throughout screening centers.

Limitation

This study has several limitations that should be considered when interpreting the findings. First, the cross-sectional design limits the ability to establish causal relationships and reflects participants’ awareness at a single point in time. Second, although the calculated sample size was statistically adequate, the use of a convenience sampling technique and restriction to individuals attending the premarital screening program in Makkah may introduce selection bias and limit the generalizability of the findings to the wider population of Makkah and other regions of Saudi Arabia. Third, the use of a self-administered questionnaire may have resulted in response bias and social desirability bias, potentially leading to overestimation of knowledge levels. Finally, certain relevant variables, such as prior exposure to educational campaigns or family history of SCD, were not fully assessed and may have influenced awareness levels.

Generalizability

It is important to take caution when generalizing the results of the study to adults undergoing premarital screening in Makkah city, as they were drawn from premarital screening centers during the study period. However, using a convenience sampling method may limit the external validity of the results. Health awareness, education level, or interest in genetic diseases may be different between those who completed the questionnaire voluntarily and the wider population. The results may not accurately represent all individuals who are planning to marry in Makkah or other regions of Saudi Arabia. The findings can be applied to similar urban populations within the Kingdom because of the large sample size and inclusion of participants from diverse demographic backgrounds.

## Conclusions

In conclusion, the study found that even though individuals participating in the premarital screening program in Makkah city have high educational levels and positive attitudes towards premarital screening, their overall knowledge and awareness of SCD is generally poor. While most participants were educated on SCD and acknowledged its importance in lowering its incidence, they did not fully comprehend its inherited nature, clinical manifestations, complications, inheritance risk, and appropriate management. Knowledge increased significantly among women, married participants, those with higher education, and those who received information from healthcare providers. Based on these findings, premarital screening programs should emphasize standardized counseling and targeted educational interventions focused on SCD. Healthcare providers should play a central role in delivering clear, simple, and culturally appropriate information regarding genetic transmission, consanguinity, and long-term complications.

## Appendices

Assessment of Knowledge and Awareness of Sickle Cell Disease Among Individuals Undergoing the Premarital Screening Program in the City of Makkah

Dear Participant,

You are invited to take part in this study, which aims to assess the knowledge and awareness of sickle cell disease among individuals undergoing the premarital screening program in the city of Makkah. We kindly ask you to complete the questionnaire and answer the questions. Please note that all responses will remain confidential and will be used for research purposes only.

**Table 6:** The Questionnaire in English

Section	Question	Response Options
Consent	Do you agree to participate in this study?	Yes/No
Demographics	Are you a resident in Makkah city?	Yes/No
Demographics	Gender	Male/Female
Demographics	Age	Under 18/18-29/30-44/45-59/60 or older
Demographics	Marital status	Single/Married
Demographics	Place of residence	Village/City
Demographics	Educational level	High school or lower/Bachelor’s degree/Postgraduate studies/Other
Knowledge (SCD)	Have you heard about sickle cell disease?	Yes/No
Knowledge (SCD)	Source of information	Television/Social media/Relatives/Healthcare provider/Other
Knowledge (SCD)	Causes of SCD	Infection/Hereditary/Immune disease/Poor lifestyle/Poor nutrition/I do not know
Knowledge (SCD)	SCD is more common in	High consanguinity/Low consanguinity/No relation/I do not know
Knowledge (SCD)	Symptoms (multiple choice)	Pallor/Fatigue/Diarrhea/Visual problems/Vomiting/Recurrent infection/Headache/I do not know
Knowledge (SCD)	Complications (multiple choice)	Chronic infection/Stroke/Hypertension/Leg ulcers/Hypoglycemia/Vascular occlusion/Increased thyroid activity/I do not know
Knowledge (SCD)	Diagnosis	Blood test/ECG/X-ray/Intestinal scope/I do not know
Knowledge (SCD)	Risk if both parents affected	100%/75%/50%/25%/0%/I do not know
Knowledge (SCD)	Crisis management	Antipyretics + fluids/Fluids + analgesics/Fluids + analgesics + oxygen/Antipyretics + analgesics/I do not know
Knowledge (SCD)	Permanent cure	Yes/No/Maybe/I do not know
Premarital Screening	Does screening include SCD?	Yes/No/Maybe
Premarital Screening	Does screening reduce SCD incidence?	Yes/No/Maybe
Attitude	If both partners are carriers, do you support continuing marriage?	Yes/No
